# A COMPREHENSIVE COMPOSITE FOR ALLOSTATIC LOAD SCORING AND RISK CATEGORIZATION FOR PREDICTIVE COMPARISONS

**DOI:** 10.1093/geroni/igad104.2764

**Published:** 2023-12-21

**Authors:** Ingrid Buller-Peralta, Sarah Gregory, Katie Wells, Georgios Ntailianis, John O’Brien, Craig Ritchie, Graciela Muniz-Terrera

**Affiliations:** University of Edinburgh, Edinburgh, Scotland, United Kingdom; University of Edinburgh, Edinburgh, Scotland, United Kingdom; University of Edinburgh, Edinburgh, Scotland, United Kingdom; University of Edinburgh, Edinburgh, Scotland, United Kingdom; University of Cambridge, Cambridge, England, United Kingdom; University of Edinburgh, Edinburgh, Scotland, United Kingdom; Ohio University, Athens, Ohio, United States

## Abstract

Introduced three decades ago, the concept of Allostatic Load (AL) describes the wear and tear of the physiological response to chronic stress, and how sustained demand overloads the systems involved, deplete their resources, and leads them to fail. Current evidence shows positive relationship between AL and risk of developing conditions such as depression, fibromyalgia, cardiovascular disease, diabetes, cancer, early cognitive decline, and dementia. However, there is only partial consensus regarding which biomarkers can compose an AL index or how to accurately score them. Current proposals debate between purely statistical cut-off points, inclusion of gender and ethnicity differences, use of clinical reference values, and scoring ongoing medication. The result is a considerable heterogeneity of methods for AL calculation and a lack of universally agreed values, leaving researchers to subjectively choose which framework suits better to their aims. Using data from the PREVENT-Dementia study (cognitively normal mid-life participants, n=700), we developed a comprehensive composite for AL scoring to generate a more accurate and biologically relevant index, that considers both previous and recent clinical evidence. By including gender and age differences, high quartile approach, and up-to-date clinical reference values, individuals were categorized as no-risk, at-risk and high-risk of AL, which will be associated with MRI brain volume measurements. Comparison to serum Thyroid-stimulating hormone (TSH) levels will serve as proxy of the neuroendocrine biomarkers originally proposed as AL primary mediators, due to its association to increased cortisol. Additional stress-related biomarkers, such as sleep quality and the neutrophil-to-lymphocyte ratio will also be assessed for associations.

